# Synergistic Effect of Co-Delivering Ciprofloxacin and Tetracycline Hydrochloride for Promoted Wound Healing by Utilizing Coaxial PCL/Gelatin Nanofiber Membrane

**DOI:** 10.3390/ijms23031895

**Published:** 2022-02-08

**Authors:** Mengxia Lin, Yuan Liu, Junwei Gao, Donghui Wang, Dan Xia, Chunyong Liang, Ning Li, Ruodan Xu

**Affiliations:** 1 Tianjin Key Laboratory of Materials Laminating Fabrication and Interface Control Technology, College of Materials Science and Engineering, Hebei University of Technology, Tianjin 300130, China; yssc1997@163.com (M.L.); liuyuan041421@163.com (Y.L.); wdh_81@163.com (D.W.); liangchunyong@hebut.edu.cn (C.L.); 2 Institute of Basic Theory for Chinese Medicine, China Academy of Chinese Medical Sciences, Beijing 100700, China; gaojwdr@163.com (J.G.); lili.li.ning@gmail.com (N.L.)

**Keywords:** coaxial nanofiber mesh, drug co-delivery, wound dressing, polycaprolactone, gelatin

## Abstract

Combining multiple drugs or biologically active substances for wound healing could not only resist the formation of multidrug resistant pathogens, but also achieve better therapeutic effects. Herein, the hydrophobic fluoroquinolone antibiotic ciprofloxacin (CIP) and the hydrophilic broad-spectrum antibiotic tetracycline hydrochloride (TH) were introduced into the coaxial polycaprolactone/gelatin (PCL/GEL) nanofiber mat with CIP loaded into the PCL (core layer) and TH loaded into the GEL (shell layer), developing antibacterial wound dressing with the co-delivering of the two antibiotics (PCL-CIP/GEL-TH). The nanostructure, physical properties, drug release, antibacterial property, and in vitro cytotoxicity were investigated accordingly. The results revealed that the CIP shows a long-lasting release of five days, reaching the releasing rate of 80.71%, while the cumulative drug release of TH reached 83.51% with a rapid release behavior of 12 h. The in vitro antibacterial activity demonstrated that the coaxial nanofiber mesh possesses strong antibacterial activity against *E. coli* and *S. aureus*. In addition, the coaxial mats showed superior biocompatibility toward human skin fibroblast cells (hSFCs). This study indicates that the developed PCL-CIP/GEL-TH nanofiber membranes hold enormous potential as wound dressing materials.

## 1. Introduction

As a global public health problem, wound healing has always been the focus of clinical treatment [1]. It is constituted of four successive physiological stages involving wound bleeding, inflammatory response, cell proliferation, and tissue remodeling [2,3,4]. The inflammatory response occurs right after the injury and lasts from 24 h to 4–6 days [5]. During this period, bacteria can easily infect the wound, leading to the aggravation of inflammation and hindering the wound healing [6,7]. The wide application of antibiotics has greatly reduced infections and promoted the development of surgery. However, with the abuse of antibiotics, drug-resistant bacteria have emerged [8], which has greatly increased the medical burden and brought substantial social and economic losses to the world [9]. Thus, a combination of multiple drugs or biologically active substances has emerged as an effective treatment method [10], which could reduce the probability of drug-resistant bacteria and effectively eradicate bacteria. The co-administration of two antibiotics may benefit from different modes of action, clearance time, and reduced toxicity of some drugs [11]. 

As a carrier for the delivering of antibiotics, nanofibers have been widely believed in the past decade to effectively prevent and reduce the risk of bacterial infections after skin injuries and wounds [12,13]. In addition, due to the large specific surface area, high porosity, high loading capacity, and similarity with skin structure and extracellular matrix, nanofibers have been widely used in topical drug delivery applications, especially in transdermal drug delivery systems [14,15,16,17]. Polycaprolactone (PCL) is considered to be a very promising precursor of electrospun cellulose nanofibers due to its good biocompatibility, mechanical properties, and ease of manufacture [18,19]. Meanwhile, PCL is a synthetic biocompatible hydrophobic polymer with extremely slow degradation rate, which could regulate the rate of drug release from scaffolds [20]. In addition, PCL has a semi-crystalline structure, which could enhance the elasticity of the scaffolds [21]. However, the synthetic PCL also has some drawbacks, such as limited cell affinity and poor hydrophilicity [22]. To improve these shortcomings, combining PCL with gelatin (GEL) with cell binding sites and biomolecular characteristics can prepare an ideal wound dressing with good biocompatibility, biodegradability, and promoted cell adhesion [23,24]. In addition, coaxial electrospinning could form two connecting layers (core–shell structure) composed of different materials, and incorporate drugs or bioactive substances into the shell–core layer [25,26]. The manufactured core–shell structure would provide improved mechanical strength, effective drug encapsulation, and sustained drug delivery [27,28].

Tetracycline hydrochloride (TH) is a hydrophilic broad-spectrum antibiotic with low toxicity and the ability to promote the adhesion of fibroblasts, which can effectively inhibit a variety of clinically relevant pathogens. Loading TH into the GEL shell layer of coaxial nanofibers could realize the releasing of large quantities in the early stage of wound healing to inhibit bacterial infection [29]. If bacterial infection occurs during wound healing, the differentiation of fibroblasts would be impaired, thus normal tissues and the immune system could be undermined [30]. In addition, the adhesion of nanofibers can lead to a massive aggregation of bacteria in a short period of time, and if these aggregated bacteria are not killed quickly, bacterial biofilms can form [31]. Therefore, it is critical to respond immediately to the presence of large numbers of bacteria in the early stages of wound healing and to prevent latent infection from developing [32]. In addition, the rapid release of early drugs can prevent the development of bacterial resistance; nevertheless, burst release during the initial period usually makes it difficult to meet the demand of long-term antibacterial requirement. The hydrophobic fluoroquinolone antibiotic ciprofloxacin (CIP), as a new broad-spectrum antibacterial drug, has strong permeability, high blood concentration, low toxicity, and is not easy for developing drug resistance [33]. Loading fat-soluble CIP into the fat-soluble PCL core layer of the coaxial nanofibers can achieve the sustained and slow release of drugs, meeting the antibacterial needs of wound healing in the inflammatory period. 

Herein, a dual drug-carrying coaxial nanofiber with different drug release rates between the inner and outer layers was prepared, playing a synergistic antibacterial effect. The coaxial PCL/GEL possesses better mechanical properties with the tensile strength of 8.92 ± 0.49 MPa compared with the uniaxial PCL (6.43 ± 0.14 MPa) and GEL (5.05 ± 0.53 MPa). Loading the drugs hardly affects the mechanical properties of the fiber membrane. The outer GEL layer makes the core–shell fiber membrane more hydrophilic (35.31°) compared with the PCL fiber (122.44°) and the contact angle would further decrease after adding CIP and TH into the core and shell of the fiber, thus quickly absorbing the wound exudate. In addition, the drugs in the inner and outer layers of the PCL/GEL nanofiber membrane are released at different stages of the wound healing with the quick release of TH for 12 h and the long-lasting release of CIP for 5 days, realizing the antibacterial synergy of drug-loaded dressings. This indicates that the nanofiber membrane can not only reduce the possibility of the formation of drug-resistant bacteria, but can also meet the antibacterial needs in the wound healing process, thus promoting the wound healing. This research may provide valuable insight into the development of a dual drug delivery system to promote wound healing.

## 2. Results and Discussion

### 2.1. Nanostructure of Coaxial Nanofibers 

**Figure 1:**
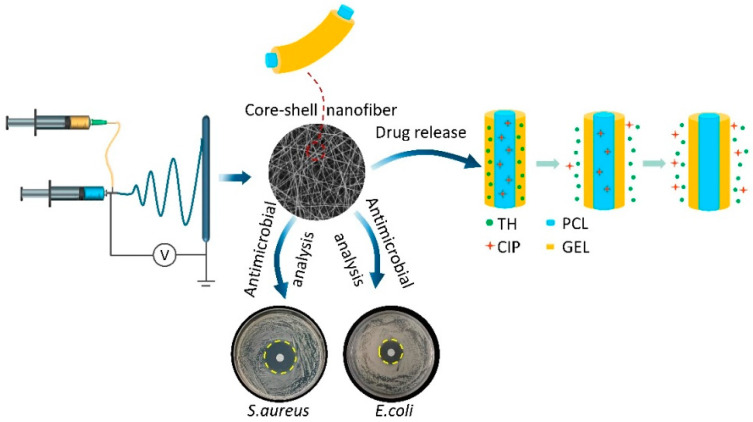
The preparation of coaxial nanofibers with co-delivering of CIP and TH antibiotics.

The preparation of the PCL-CIP/GEL-TH core–shell nanofibers is shown in Figure 1. Briefly, the PCL loaded with CIP acts as the core layer, while the GEL loaded with TH acts as the shell layer, and the dual drug-loaded nanofiber membranes were prepared by coaxial electrospinning. The co-delivery of two antibiotics with different modes of action could satisfy the antibacterial needs at different stages of the wound healing process.

**Figure 2:**
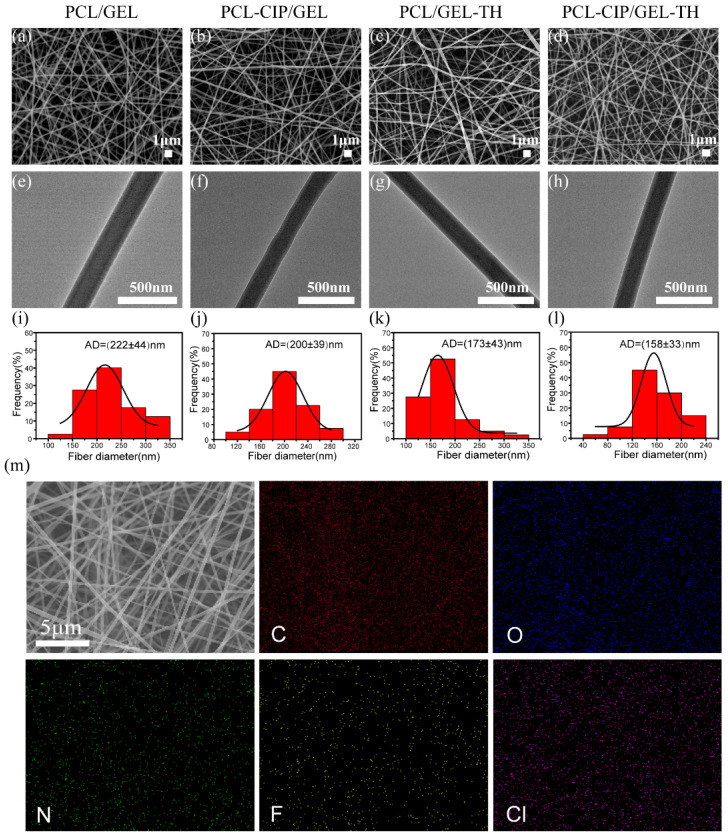
(**a**–**d**) SEM images of different coaxial nanofiber membranes; (**e**–**h**) TEM images of different core–shell nanofibers; (**i**–**l**) diameter distribution of different nanofibers; (**m**) EDS mapping of the PCL-CIP/GEL-TH nanofibers.

The nanostructure of the prepared core–shell nanofiber membrane is shown in Figure 2. The scanning electron microscopy (SEM) images (Figure 2a–d) show that the nanofibers were randomly distributed and the fiber surfaces were smooth with no cracks, pores, or beads. The transmission electron microscopy (TEM) images (Figure 2e–h) confirm the core–sheath structure of the nanofibers. A clear boundary between the PCL core layer and the GEL shell layer was observed and the core–shell structure of the fibers was not affected by the drug loading. The diameters of the PCL/GEL, the PCL-CIP/GEL, the PCL/GEL-TH, and the PCL-CIP/GEL-TH fibers (Figure 2i–l) were 222 ± 44 nm, 200 ± 39 nm, 173 ± 43 nm, and 158 ± 33 nm, respectively. The fibers loaded with drugs are thinner than those without drugs, which may be because the addition of drugs increases the conductivity of the solution and the electric field force of the spinning jet, resulting in finer fibers [34,35]. In addition, no phase separation occurred after the fibers were loaded with the drug, indicating that the CIP and TH drugs were uniformly dispersed in the polymer matrix. To prove that the CIP and TH were successfully loaded into the coaxial polymer, an energy dispersive spectroscopy (EDS) was carried out. Figure 2m shows the EDS mapping of the PCL-CIP/GEL-TH fiber, in which the characteristic elements of CIP (fluorine, F) and TH (chlorine, Cl) are distributed evenly on the PCL-CIP/GEL-TH nanofibers. These results provide strong evidence that TH and CIP are successfully loaded into the PCL-CIP/GEL-TH nanofiber.

### 2.2. Fourier Transform Infrared Spectroscopy (FTIR)

**Figure 3:**
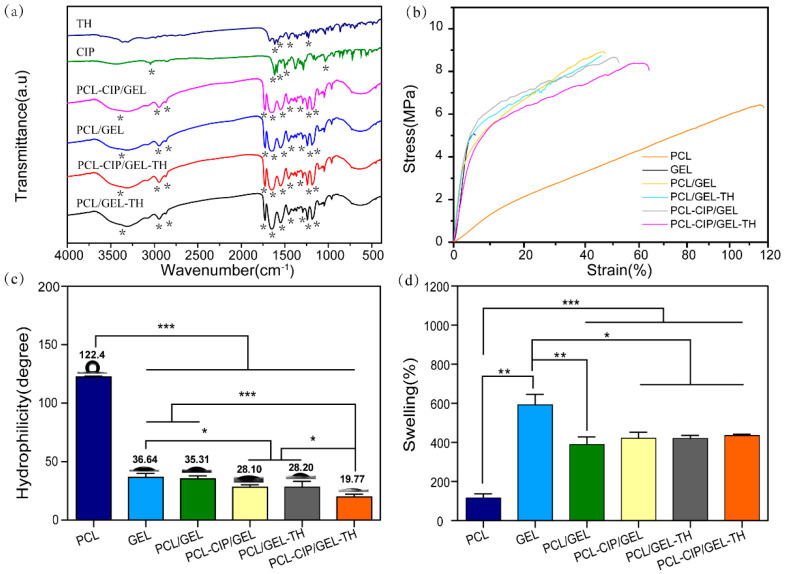
Physico-chemical characterization of different nanofibers. (**a**) FTIR spectra; (**b**) stress–strain curves; (**c**) water contact angles; (**d**) swelling behaviors. (*n* = 3); *—*p* < 0.05; **—*p* < 0.01; ***—*p* < 0.001.

Figure 3a shows the FTIR spectra of different samples. The results show that the coaxial PCL/GEL scaffold possesses characteristic peaks at 1647 cm^−1^, 1548 cm^−1^, 2949 cm^−1^, and 2865 cm^−1^, corresponding to the amide Ⅰ and amide Ⅱ bonds, asymmetric CH_2_ stretching, and symmetric CH_2_ stretching of GEL, respectively [36]. Meanwhile, the coaxial PCL/GEL scaffolds also display characteristic peaks of PCL at 1725 cm^−1^ (carbonyl group), 1240 cm^−1^ (COC stretching), and 1165 (1191) cm^−1^ (symmetric COC stretching) [37,38]. All the core–shell nanofibers present both the characteristic peaks of PCL and GEL, confirming the successful combination of both PCL and GEL in the coaxial nanofibrous membranes. The characteristic peaks of CIP at 1617 cm^−1^ and 3049 cm^−1^ are caused by the stretching vibration of the C=O bond and the OH bond, while the peak at 1282 cm^−1^ is due to the coupling of the stretching vibration of the CO bond and the deformation vibration of the OH bond in the carboxylic acid. In addition, the characteristic peaks of CIP at 1589 cm^−1^ and 1499 cm^−1^ are caused by the stretching vibration of the benzene ring [39,40]. The distinct peaks of TH at 1613 cm^−1^ and 1579 cm^−1^ correspond to the C=O (A and C) ring and NH_2_ amide bond [41]. However, no characteristic peaks of CIP and TH were observed for the drug-loading coaxial fiber mats, maybe caused by the low amounts of CIP and TH in the nanofibers or maybe the characteristic peaks of CIP and TH were covered by that of PCL and GEL. [42,43] However, the successful loading of CIP and TH was confirmed by the EDS results (Figure 2m).

### 2.3. Mechanical Properties

The mechanical performances of wound dressing should match the properties of human skin, which has the tensile strength, Young’s modulus, and elongation at break of 1–32 MPa, 2.9–150 MPa, and 17–207%, respectively, [44] to avoid further injury. Figure 3b demonstrates the mechanical properties of the different electrospun scaffolds. The results show that the tensile strength, elongation at break, and Young’s modulus of GEL were 5.05 ± 0.53 MPa, 5.07 ± 0.78%, and 118.66 ± 0.49 MPa, respectively, while that of PCL were 6.43 ± 0.14 MPa, 115.53 ± 7.77%, and 12.77 ± 0.02 MPa, respectively. Compared with uniaxial GEL, the tensile strength (8.92 ± 0.49 MPa), and elongation at break (47.08 ± 5.03%) of coaxial nanofiber PCL/GEL are significantly improved, due to the semi-crystalline structure of PCL enhancing the elasticity of the scaffold. Meanwhile, compared with uniaxial PCL, the tensile strength (8.92 ± 0.49 MPa) and Young’s modulus (124.28 ± 0.47 MPa) of coaxial PCL/GEL were increased. This may be because during the coaxial electrospinning process, the shell spinning solution is stretched at a high speed under the action of the electric field force, and the core spinning solution interface produces a shearing effect, resulting in the orientation of the polymer molecular chains in the core spinning solution along the fiber axis, thus improving the mechanical strength. Meanwhile, the shell material has a certain protective effect on the core layer material, which can reduce or even inhibit the Rayleigh instability of the core layer jet [45]; therefore, the core layer polymer material can rearrange and crystallize better, improving the elastic modulus and tensile strength of the coaxial nanofibers [46]. In addition, the loading of drugs hardly affects the mechanical properties of the fiber membrane; therefore, the prepared core–hell nanofiber could provide good mechanical properties for wound healing [47,48].

### 2.4. Contact Angle of Core–Shell Nanofibers

The hydrophilicity of nanofiber membranes as a wound dressing affects cell adhesion and proliferation. The water contact angles of the fiber membranes were measured to evaluate their hydrophilicity (Figure 3c). The results show a hydrophobic surface of the PCL with an average contact angle of 122.44°, which is not beneficial to wound healing. However, GEL is hydrophilic (36.64°), which can be used to improve the hydrophilicity of PCL; therefore, by combining the hydrophilic GEL with the hydrophobic PCL the contact angle of the coaxial nanofiber PCL/GEL decreases to 35.31°. Adding CIP and TH into the core and/or the shell of the nanofiber would further reduce the contact angles of the coaxial nanofibers, which may be because a small part of the drugs that were distributed on the fiber surface were beneficial to the contact between the water and the membrane surface.

### 2.5. Water Absorption

The good water absorption ability of the wound dressing would help absorb the wound exudate [49]. Figure 3d shows the swelling behavior of different nanofibers. Compared with uniaxial PCL (116.97%) the water absorption abilities of all the coaxial nanofibers were improved, and the corresponding water absorption values of PCL/GEL, PCL-CIP/GEL, PCL/ GEL-TH, and PCL-CIP/ GEL-TH were 389.64%, 421.63%, 410.19%, and 435.00%, respectively. However, the water absorption abilities of the coaxial nanofibers were still lower than that of the uniaxial GEL nanofibers (592.53%). This is because wrapping the hydrophobic PCL core layer with the hydrophilic GEL layer possessing a lot of hydrophilic groups on the surface would form hydrogen bonds when contacting the coaxial nanofiber with water molecules, thus significantly improving the water adsorption abilities of the nanofiber [50]. In addition, the coaxial nanofiber only possess a portion of GEL, hence the water adsorption ability is still lower than that of the pure GEL nanofibers. The improvement of water absorption makes the coaxial fiber membrane more in line with the needs of wound dressings.

### 2.6. In Vitro Drug Release

**Figure 4:**
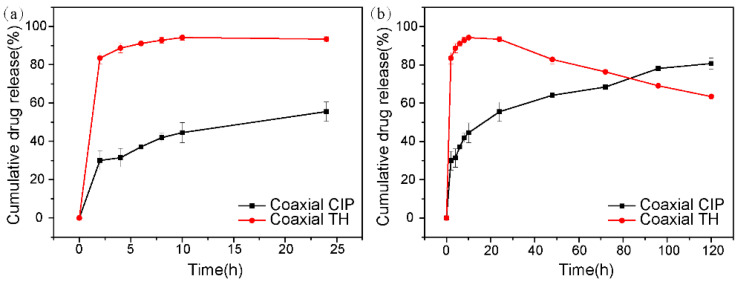
In vitro drug release of CIP and TH at different periods. (**a**) 24 h; (**b**) 120 h.

The core–shell dual drug-loaded fiber mat can deliver different drugs in chronological order to satisfy the needs of different wound healing stages. Figure 4 shows the in vitro CIP and TH release curves measured in PBS (pH = 7.4) at 37 °C. It shows that the release of TH is fast, reaching 83.51% within 2 h, and almost completed within 10 h (Figure 4a). This is because the hydrophilic GEL is unstable after encountering the water molecules, and the fiber structure is destroyed, resulting in the rapid release of the TH. However, the release profile of CIP was different to TH. Specifically, the cumulative release rate of CIP reached 29.99% in the first 2 h, and then slowly reached 55.53% at 24 h. In the following 4 days, the release rate of CIP was even slower and the accumulative released amount of CIP after 5 days was found to be 80.71% (Figure 4b). This is because PCL is a synthetic biocompatible hydrophobic polymer with extremely slow degradation rate, thus the releasing rate of CIP from the scaffold is reduced. The synergistic effect of the two drugs can achieve free and rapid release of TH in the early stages of the wound in order to combat wound infection, and then gradually release CIP to kill the bacteria in the later stages in order to meet the needs of the wound healing in the inflammatory period.

### 2.7. Antibacterial Activity Analysis

**Figure 5:**
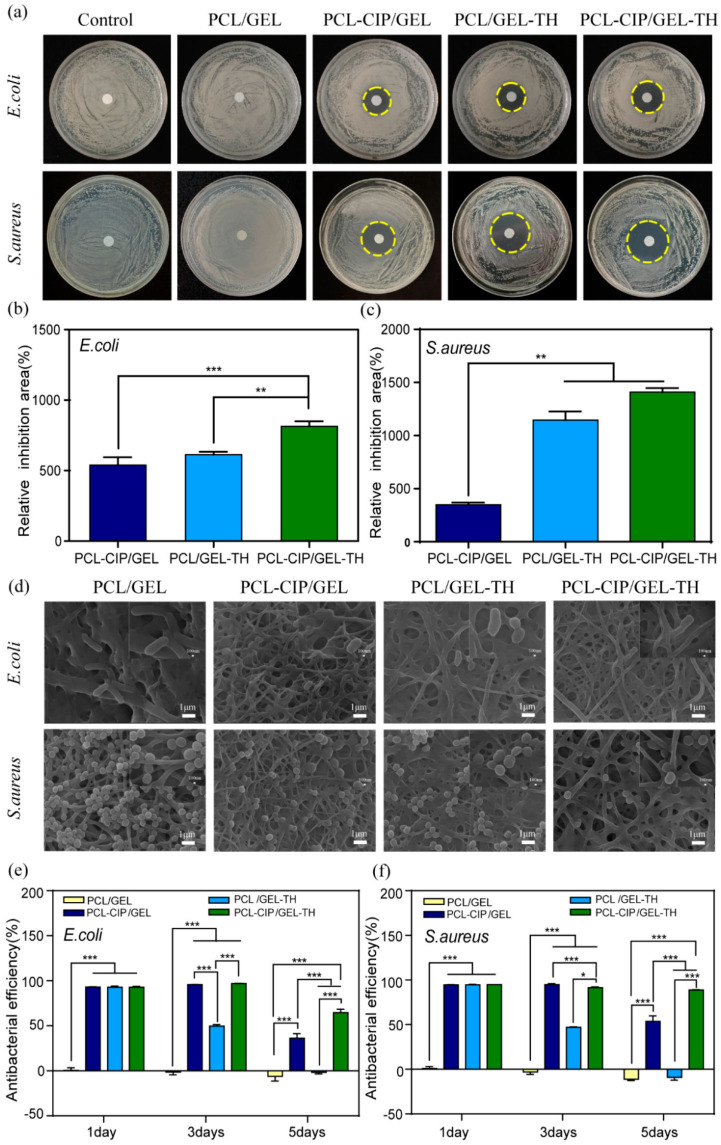
Antibacterial activity of coaxial nanofibers against *E. coli* and *S. aureus*. (**a**) Inhibition zone assay; (**b**,**c**) bar diagrams of antibacterial efficacy of different membranes (*n* = 3); (**d**) SEM images of the morphologies of *E. coli* and *S. aureus* on different nanofibers; (**e**,**f**) the long-lasting antibacterial effects evaluation of different nanofibers by CCK-8 assay (*n* = 3). *—*p* < 0.05; **—*p* < 0.01; ***—*p* < 0.001.

Bacterial activity at the wound site may cause inflammation and infection, thus delaying the wound healing process [51]; therefore, the antibacterial activity of wound dressing plays a crucial role in the healing process of skin wounds. To evaluate the antibacterial performance of the nanofiber mats, the disc diffusion method was used to quantify the size of the inhibition zone (Figure 5a). The results show that the PCL/GEL coaxial nanofiber membrane without drug loading did not show an obvious inhibition zone for both *Escherichia coli* (*E. coli*) and Staphylococcus aureus (*S. aureus*), indicating no antibacterial activities. The diameters of the inhibition zone of PCL-CIP/GEL, PCL/GEL-TH, and PCL-CIP/GEL-TH against *E. coli* were 20.22 ± 0.72 mm, 21.37 ± 0.25 mm, and 24.18 ± 0.40 mm, respectively, while those against *S. aureus* were 16.95 ± 0.28 mm, 28.23 ± 0.65 mm, and 31.07 ± 0.28 mm, respectively. In addition, the relative inhibition area of PCL-CIP/GEL, PCL/GEL-TH, and PCL-CIP/GEL-TH against *E. coli* were 539.70 ± 45.89%, 613.51 ± 16.71%, and 813.88 ± 29.99%, respectively, while those against *S. aureus* were 348.76 ± 14.56%, 1146.03 ± 57.79%, and 1408.80 ± 27.19%, respectively (Figure 5b,c). Obviously, the fiber membranes loaded with a single drug (CIP or TH) present a significant inhibition zone against both *E. coli* and *S. aureus*, indicating an inhibitory effect of drugs on bacteria. The diameter of the antibacterial zone of the dual drug-loaded fiber membrane is larger than that of the single drug delivery system, indicating a more significant antibacterial effect of the co-administration of two drugs. The antibacterial effect of the prepared nanofiber membrane is similar to that reported by Sang et al. [52]. However, the amount of drug loading in the shell layer is one hundred times lower and in the core layer it is ten times lower for our study. This study also showed that the prepared wound dressing had a greater bactericidal effect on Gram-positive *S. aureus* than Gram-negative *E. coli*. This is because the outer impermeable membrane of *E. coli* protects the cell wall peptidoglycans, hence it becomes generally more resistant to antibacterial drugs [53]. The morphology of *E. coli* and *S. aureus* cultured on different nanofiber surfaces for 12 h were examined by SEM (Figure 5d). It shows both *E. coli* and *S. aureus* incubated with four different nanofibers remaining intact, with smooth cell membranes, and in their typical morphology. In addition, the amount of *E. coli* and *S. aureus* on the PCL/GEL nanofiber mat is significantly larger than on the drug-loading substrates (PCL-CIP/GEL, PCL/GEL-TH, PCL-CIP/GEL-TH), which indicates their fine antibacterial effect.

A CCK-8 test of bacteria was carried out to further verify the long-term bacteriostatic effect of the coaxial nanofiber membrane. Figure 5e,f show the nanofiber membranes incubated in the bacterial solution for 1, 3, and 5 days. The PCL/GEL coaxial nanofiber without drug loading had no antibacterial effect with low antibacterial efficiency during the 5 days. The other three drug-loaded nanofibers (PCL-CIP/GEL, PCL/GEL-TH, and PCL-CIP/GEL-TH) exhibited the high antibacterial efficiency at day 1 due to their excellent antibacterial effects by the large amount of drug releasing in the early stage. On day 3, the antibacterial efficiency of the drug-loaded nanofibers was still high compared to the PCL/GEL coaxial nanofiber. For the three drug-loaded nanofibers, the antibacterial efficiency of PCL/GEL-TH was relatively lower than PCL-CIP/GEL and PCL-CIP/GEL-TH, due to the fast releasing of TH in the shell layer. On day 5, the antibacterial efficiency of PCL/GEL-TH is comparable with that of PCL/GEL, indicating no antibacterial effect, while the antibacterial efficiency of PCL-CIP/GEL was relatively higher than that of PCL/GEL, but was lower than that on day 3, indicating the limited antibacterial effect for 3 days. For the dual drug-loaded PCL-CIP/GEL-TH, the antibacterial efficiency was still high at day 5, indicating the long-lasting antibacterial effect. Again, the PCL-CIP/GEL-TH had a greater bactericidal effect on Gram-positive S. aureus than Gram-negative E. coli. These results indicate that the coaxial nanofiber PCL-CIP/GEL-TH has high-efficiency and long-lasting antibacterial properties.

### 2.8. Cytotoxicity Assay

**Figure 6:**
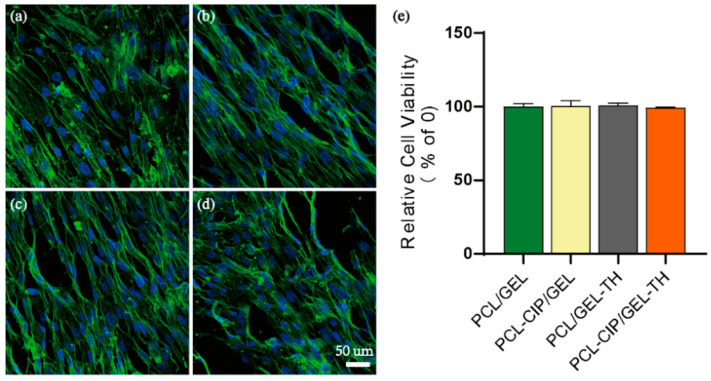
(**a**–**d**) The fluorescent images and (**e**) the viability of hSFCs cultured for 24 h on different samples.

The wound dressing should not be cytotoxic [54]. The cytocompatibility of different nanofibrous scaffolds towards the human skin fibroblast cells (hSFCs) was visualized by confocal laser scanning microscopy (FV3000, Olympus Cooperation, Tokyo, Japan) after a 1 day culture on the scaffolds (Figure 6). It is shown that the hSFCs adhered and spread widely on all scaffolds, which could be attributed to the fibrous structural protein nature of collagen composed on the surface of the scaffolds. On the other hand, the group containing antibiotics showed more cell nucleus, which may contribute to inducing bacterial death and promoting cell growth to some degree compared to the group without antibiotics. In parallel, the cell viability of the hSFCs was conducted at day 1 using an MTS assay, showing no statistical differences amongst all groups, confirming the biocompatibility of all the groups.

## 3. Materials and Methods

### 3.1. Material

The PCL (average Mn = 80,000) and GEL from porcine skin (Type A) were purchased from Sigma-Aldrich, St. Louis, MO, USA. The 2,2,2-Trifluoroethanol (TFEA) was purchased from Macklin Biochemical Technology Co., Ltd., (Shanghai, China). The CIP and TH were purchased from Aladdin Reagent Co., Ltd., (Shanghai, China). The glutaraldehyde was supplied by Fuchen Chemical Reagent Co., Ltd., (Tianjin, China). The count assay Kit-8 (CCK-8) was procured from Soleibao Technology Co., Ltd., (Beijing, China). The glutaraldehyde fixation solution was procured from Leagene Biotech. Co., Ltd., (Beijing, China).

### 3.2. Preparation of Coaxial PCL/GEL Nanofibers

The PCL was dissolved in TFEA, forming a shell solution of PCL (9% *w*/*v*), while the GEL was dissolved in TFEA and deionized (DI) water (3:1, *v*/*v*), forming a core solution of GEL (10% *w*/*v*). The two solutions were stirred overnight with magnetic stirrers. Coaxial needles (inner 21 G, outer 15 G) were utilized for coaxial electrospinning. A constant voltage of 8.5 kV was applied, and the distance between the needle and the receiver was 15 cm. The speed of the syringe with the shell solution and the inner solution was 0.04 mm/min and 0.02 mm/min, respectively. To prepare the drug-loading nanofibers, the CIP and/or TH (1% *w*/*w*) were added into the solution and stirred well. The coaxial PCL-CIP/GEL and PCL/GEL-TH nanofiber membranes containing a single drug and PCL-CIP/GEL-TH containing two drugs were obtained. The prepared core–shell nanofibers were then cross-linked by the glutaraldehyde (2.5% *w*/*w*) in order to obtain better water stability. The uniaxial PCL fiber membrane and the GEL fiber membrane were also prepared for comparison.

### 3.3. Characterization of Different Fiber Membranes

#### 3.3.1. Nanostructure and Composition

The morphology of nanofibers was observed by SEM (JSM-7100F, JEOL Ltd., Tokyo, Japan) with an acceleration voltage of 10 kV after sputter coating the samples with gold. The coaxial structure of the nanofibers was determined by TEM (Talos F200S, FEI, Hillsboro, OR, USA). For TEM observation, nanofibers were directly deposited onto copper grids and were observed after drying the copper mesh. FTIR (TENSOR 27, Bruker, Karlsruhe, Germany) and EDS element mapping (JSM-7100F, JEOL Ltd., Tokyo, Japan) was used to determine whether or not CIP and TH were encapsulated in the nanofibers.

#### 3.3.2. Contact Angle and Water Absorption

The hydrophilicity of scaffolds was evaluated by calculating the water contact angle at room temperature (RT) (JC2000DM). Nanofibers (10 mm × 10 mm) were attached to glass slides and apparent contact angles were determined by the sessile drop method. The droplet size was set for 3 L and the results were obtained after DI water was dropped freely onto the surfaces of the samples (*n* = 3). The samples were cut into a membrane with a size of 10 mm × 10 mm, then the samples were placed in PBS (pH = 7.4) and incubated at 37 ℃. The scaffolds were immediately weighted after removing the surface water of samples. The water absorption rate (AR_water_) was determined by the equation of AR_water_ = (M − M_0_)/M_0_ × 100%, where M_0_ and M are the weights of the nanofibers before and after immersion in the PBS solution, respectively.

#### 3.3.3. Mechanical Properties

The mechanical properties of the electrospun fiber membranes were tested by a tensile and compression testing machine (HY-0580) equipped with a load of 20 N. The tensile properties of the sample were tested under the stretching speed of 5 mm/min. All the experiments were performed in triplicate for each time.

#### 3.3.4. In Vitro Drug Release 

The drug-loaded nanofibers (20 mg) were immersed in 10 mL PBS and incubated at 37 °C with constant shaking at 100 rpm. At a specific time point, the drug-released solution (3 mL) was taken out to measure the absorbance of TH and CIP at the wavelength of 361 nm and 277 nm by UV–visible spectroscopy. Then, the same amount of fresh PBS buffer was added to maintain the same release environment. The concentration and the cumulative release of the drugs were calculated through the standard curve, and the release rate was calculated based on the initial added drug amount. Then, the release rate versus time was plotted accordingly. Since one (277 nm) of the two characteristic peaks of TH (277 nm and 361 nm) coincides with the characteristic peak of CIP (277 nm), the core–shell nanofibers loaded with one drug each time was used to detect the in vitro drug release.

#### 3.3.5. Antibacterial Activity

The antibacterial activity of the nanofiber mat was determined by the inhibition zone against Gram-positive *S. aureus* and Gram-negative *E. coli*. The nanofiber membranes (diameter 8 mm) containing antibiotics were placed on agar plates containing bacteria (10^6^ CFU/mL). The plates were incubated at 37 °C for 12 h and then the inhibition zone (diameter) was determined. The relative inhibition area was calculated according to the following equation: the relative inhibition area (%) = (area of inhibition zone−nanofiber mat area)/nanofiber mat area × 100%. Each test was repeated three times and the results were expressed as mean ± standard deviation. The effects of the nanofiber membranes on *E. coli* and *S. aureus* were analyzed by SEM. In brief, 10^6^ CFU/mL of *E. coli* and *S. aureus* treated with the different nanofiber membranes for 12 h were incubated at 37 °C. The obtained nanofiber membranes were washed with PBS and fixed with 2.5% glutaraldehyde for 6 h at 4 °C. Then, the samples were dehydrated by a series of ethanol with different concentrations. Finally, the morphologies of the different samples were observed by SEM.

The long-lasting antibacterial effect was evaluated by CCK-8. Briefly, the prepared nanofiber membranes were placed into a 48-well tissue culture plate. 500 μL of bacterial suspension containing 10^5^ CFU/mL bacteria was added into each well and incubated for 5 days at 37 °C. The sterile LB broth was replaced every 24 h. The bacteria proliferation was evaluated using CCK-8 at predetermined time intervals.

#### 3.3.6. Cell Culture and Cell Seeding on Scaffolds

The hSFCs were purchased from the BeNa Culture Collection (BNCC, Beijing, China). Different nanofibers (PCL/GEL, PCL-CIP/GEL, PCL/GEL-TH, and PCL-CIP/GEL-TH) were cut into wafers with a diameter of 8 mm and irradiated on both sides of the wafers for 30 min under UV light. hSFCs with the density of 4.5 × 10^4^ were cultured in a cell growth medium containing DMEM with high glucose (Gibco, Thermo Fisher, Waltham, MA, USA) supplemented with L-glutamate and sodium pyruvate additive, and 10% FBS (Gibco, Thermo Fisher, Waltham, MA, USA). The cells were grown at 37 °C in a humidified atmosphere of 5% CO_2_. The cells were enzymatically treated with trypsin for passaging every 5–7 days. 

#### 3.3.7. In Vitro Cytotoxicity Evaluation

The cell viability of hSFCs on different nanofibrous scaffolds was performed using the CellTiter96^®^ AQueous One Solution (Promega, Madison, WI, USA). In brief, the cell culture medium was removed after a 24 h culture, four groups of scaffolds were immersed with a diluted MTS solution (30 L MTS reagent in 300 L fresh culture medium) in each well, and incubated for 4 h. The same volumes of culture medium and MTS reagent without cells were also incubated as the background. 100 L solution was transferred into a 96-well plate, and the absorbance at 490 nm was measured for each well as above.

#### 3.3.8. Immunocytochemistry

Cellular morphology was visualized after the 24 h culture using fluorescence microscopy. Briefly, the cell-laden constructs were fixed with 4% paraformaldehyde in PBS for 10 min at RT. After rinsing with PBS three times, the samples were placed in a permeabilization solution with 0.2% Triton X-100 for 10 min and rinsed again three times with fresh PBS. The cell-laden constructs were then blocked with 1% BSA in PBS for 1 h at RT. Immunostaining was performed with Alexa Fluor 488 Phalloidin (Gibco, Thermo Fisher, Waltham, MA, USA) and DAPI (Sigma-Aldrich, Missouri, USA) to visualize the F-actin and nuclei, respectively. The cell-laden constructs were visualized using an Olympus FV3000 laser confocal microscope.

### 3.4. Statistics

The quantitative results are shown as the means ± standard deviation of mean. The statistical analyzes were performed using the Student’s *t*-test to compare different data groups. The *p* < 0.05 was considered statistically significant.

## 4. Conclusions

In summary, the nanofibers with coaxial structure were prepared by using electrospinning technology to realize the sequential dual-drug release according to the needs of the different wound healing periods. The nanofibers have a good surface morphology and a clear core–shell structure. The presence of the outer layer of gelatin improves the hydrophilicity and the water absorption of the coaxial nanofibers. The inner and outer layers of fibers are respectively loaded with two antibiotics with different hydrophilicity and hydrophobicity, which are released in sequence. The rapid release of the hydrophilic drug TH and the slow release of the hydrophobic drug CIP are combined to meet the antibacterial needs of the different stages of wound healing. The antibacterial activity experiment showed that the co-loading of the two antibiotics inhibited *E. coli* and *S. aureus* better than the single-loaded one. The mechanical properties of the nanofibers meet the requirements of wound dressings. The dual drug-loaded coaxial nanofiber membrane synthesized in this study is a potential ideal antibacterial dressing for wound healing.

## Data Availability

The authors declare that all data supporting the findings are available within the paper or are available from the authors upon request.
